# REAC RGN-AR treatment modulates adipogenic differentiation in adipose tissue-derived stem cells

**DOI:** 10.1038/s41598-026-35204-2

**Published:** 2026-01-08

**Authors:** Sara Cruciani, Salvatore Rinaldi, Vania Fontani, Margherita Maioli

**Affiliations:** 1 Department of Biomedical Sciences, University of Sassari, 07100 Sassari, Italy; 2 Present Address: Department of Reparative and Regenerative Medicine, Rinaldi Fontani Institute, 50144 Florence, Italy; 3 Present Address: Research Department, Rinaldi Fontani Foundation, 50144 Florence, Italy

**Keywords:** Biotechnology, Molecular medicine

## Abstract

Adipose tissue-derived stem cells (ADSCs) possess multipotent differentiation potential and significant immunomodulatory properties, making them valuable in regenerative medicine. However, their adipogenic differentiation can lead to triglyceride accumulation, chronic inflammation, and metabolic dysfunction. This study evaluated the effects of Radio Electric Asymmetric Conveyer (REAC) technology tissue optimization regenerative adipogenesis reprogramming (TO RGN-AR) on ADSC differentiation, focusing on its ability to preserve stemness, suppress adipogenesis, and promote beneficial phenotypes. REAC TO RGN-AR treatment significantly increased the expression of stemness-related genes (Oct-4, Sox2, and Nanog) while downregulating the expression of adipogenic markers (PPAR-γ, LPL, and ACOT2). Additionally, REAC TO RGN-AR treated cells presented a phenotypic shift toward beige adipocytes, characterized by increased TMEM26 expression and reduced ASC-1 expression. These findings underscore the novelty of using REAC TO RGN-AR to modulate cellular endogenous bioelectrical activity, presenting a noninvasive and operator-independent approach to enhance ADSC-based therapies. This work highlights the potential of this treatment to address metabolic disorders and chronic inflammation while advancing regenerative medicine.

## Introduction

The introduction does not include a heading and should expand on the background of the topic, typically including in-text citations^1^. Adipose tissue-derived stem cells (ADSCs) have emerged as promising tools in regenerative medicine because of their abundance, ease of isolation, and differentiation potential^1,2^. As a subset of mesenchymal stem cells (MSCs), ADSCs are capable of differentiating into multiple lineages^3^, including adipogenic^4^, osteogenic, and chondrogenic lineages^5^, and exhibit significant immunomodulatory properties. These features position ADSCs as key candidates for therapies targeting tissue regeneration^6^, immune modulation^7,8^, and metabolic dysfunction^8^.

Despite their versatility, ADSC differentiation into adipogenic lineages poses challenges for therapeutic applications^9^, particularly in conditions associated with metabolic disorders^10^. Adipogenic differentiation involves lipid accumulation and the activation of transcriptional regulators such as peroxisome proliferator-activated receptor gamma (PPAR-γ)^11^ and CCAAT-enhancer-binding proteins (C/EBPs)^12^, leading to the formation of mature adipocytes^4^. This process is closely associated with metabolic dysfunctions, including insulin resistance and chronic low-grade inflammation^13^, which are hallmarks of obesity-related diseases such as type 2 diabetes and cardiovascular disorders^4^.

White, brown, and beige adipocytes differ in morphology, function, and metabolic relevance. White adipocytes mainly store triglycerides and contribute to the release of pro-inflammatory adipokines. Brown adipocytes, characterized by abundant mitochondria and the expression of uncoupling protein 1 (UCP1), dissipate energy as heat. Beige adipocytes are inducible within white fat depots and exhibit thermogenic and anti-inflammatory properties, representing a metabolically favorable phenotype. Understanding and modulating the shift between these cell types has become crucial for therapeutic approaches addressing metabolic and inflammatory pathologies.

The potential to modulate ADSC differentiation while preserving their stemness is of significant clinical interest. Endogenous bioelectrical modulation, an emerging field in regenerative medicine, has demonstrated promise in influencing cellular behavior through the restoration of endogenous bioelectrical homeostasis. Radio electric asymmetric conveyer (REAC) technology represents a novel approach in this domain, delivering asymmetrically conveyed radio electric fields to noninvasively modulate cellular activity^14–16^. This asymmetric conveyance ensures the selective targeting of the endogenous bioelectrical imbalances, spares unaffected areas, and promotes cellular responses that restore physiological homeostasis.

Previous REAC studies have demonstrated that specific exposure protocols can prime differentiation and reprogramming processes. For example, REAC stimulation enhanced cardiac, neuronal, and skeletal muscle gene expression in mouse embryonic stem cells^17^; directly reprogrammed human fibroblasts toward these same lineages without chemical or genetic vectors^18^; and optimized multipotency and lineage commitment in human adipose-derived stem cells obtained via Lipogems^19^. These findings consolidate the methodological foundation and biological rationale of the present study.

Research has shown that Specific therapeutic protocols of REAC technology are capable of promoting tissue regeneration^20–22^, reduces inflammation^23–25^, and enhances metabolic responses^26,27^.

The latter include improvements in oxidative metabolism, mitochondrial efficiency, and restoration of anabolic–catabolic equilibrium, as previously documented in REAC-treated cellular systems.

This study investigated the impact of REAC tissue optimization regenerative adipogenesis reprogramming (TO RGN-AR) on ADSCs, focusing on its ability to modulate molecular markers and phenotypic outcomes associated with adipogenic differentiation.

By exploring the early stages of adipogenic commitment and the induction of beige phenotypic traits under REAC-induced bioelectrical modulation, this work aims to provide new mechanistic insight into how restoring bioelectrical homeostasis may counteract metabolic dysfunction and promote regenerative processes.

## Methods

### Cell isolation and culture

ADSCs were isolated after written informed consent was obtained from the subcutaneous adipose tissue of male and female patients during general surgery procedures, who were not diagnosed with obesity, diabetes, or other related metabolic diseases (n = 6, age = 45 ± 15 years, BMI: 22 ± 3 kg/m2). The study was approved by the Review Board of the Human Studies Ethics Committee of Sassari (n° ETIC 240I/CE 26 July 2016, Ethical Committee, ASL Sassari) All experimental procedures were performed in accordance with relevant guidelines and regulations, including the Declaration of Helsinki and institutional protocols. Samples of adipose tissue were washed in PBS (Euroclone, Milan, Italy), minced into small fragments and digested with type I collagenase solution for 1 h at 37 °C (Gibco Life Technologies, Grand Island, NY, USA). ADSCs were immunomagnetically separated from the total population isolated and characterized by flow cytometry, as previously described^28^. The cells were then grown in basic growth medium consisting of Dulbecco’s modified Eagle’s medium (DMEM) (Life Technologies Grand Island, NY, USA) supplemented with 20% fetal bovine serum (FBS) (Life Technologies, Grand Island, NY, USA), 200 mM L-glutamine (Euroclone, Milan, Italy), and 200 U/mL penicillin and 0.1 mg/mL streptomycin (Euroclone, Milan, Italy).

### Adipogenic differentiation

Adipogenic differentiation was induced via specific adipogenic differentiation medium (DM) (StemPro adipocyte differentiation medium; Gibco Life Technologies, Grand Island, NY, USA). Adipogenesis was induced for a total of 14 days and assessed through immunofluorescence analysis of specific adipogenic markers. The experimental groups included untreated cells (negative control), cells exposed to DM (positive control), and cells exposed to both DM and TO RGN-AR.

### REAC treatment protocol

The REAC TO-RGN-AR treatment was performed using the REAC BENE mod. 110 medical device (ASMED, Scandicci, Italy), which is specifically designed to deliver asymmetrically conveyed radio electric fields for bioelectrical modulation. Asymmetric Conveyor Probes (ACPs) were positioned inside the culture plates, ensuring appropriate contact with the culture medium.

The device emits extremely weak radio electric fields, which interact with cellular structures to target and modulate areas of altered endogenous bioelectrical activity. The treatment protocol used in this study is a proprietary, manufacturer-defined sequence of impulses designed for tissue optimization and adipogenic reprogramming. The device parameters are factory pre-set and cannot be altered by the operator, ensuring reproducibility and standardization across all experiments.

Control groups were cultured under identical environmental and experimental conditions but were not exposed to the radio electric field. This methodological approach reproduces the same exposure setup described in previous REAC cellular studies^17–19^, which demonstrated consistent and specific bioelectrical modulation effects on stem and somatic cells.

The treatment was applied continuously for 72 h under controlled conditions (37 °C, 5% CO₂), ensuring consistent cellular viability and experimental reproducibility. The cells were then maintained in culture for up to 14 days in the presence of basic growth- or differentiation-conditioned media. The device parameters, fixed by the manufacturer, preclude operator adjustments, standardizing treatment across replicates. The control groups were cultured under identical environmental conditions without exposure to the radio electric field, which served as a baseline for comparison. This approach underscores the unique ability of asymmetrically conveyed radio electric fields to noninvasively modulate cellular processes, warranting further investigation into their molecular effects.

### Molecular analysis

Quantitative RT‒qPCR was used to evaluate the expression of stemness-related Oct-4, Sox2, and Nanog^29^ and adipogenic-related (ACOT2^30^, LPL^31^, aP2^31^, PPAR-γ^11^ and UCP1^32^) markers. Gene expression analysis was performed after 14 days of culture. Total RNA was extracted via a ChargeSwitch kit (Thermo Fisher Scientific, Grand Island, NY, USA). Approximately 1 µg of total RNA from each sample was quantified via a NanoDrop™ One/OneC microvolume UV‒Vis spectrophotometer (Thermo Fisher Scientific, Grand Island, NY, USA) and used for qPCR. Real-time quantitative PCR was performed via Luna Universal qPCR Master Mix (Euroclone, Milan, Italy) in a CFX Thermal Cycler (Bio-Rad, Hercules, CA, USA).

Each reaction was performed in triplicate for each experimental condition, and the mean of the technical replicates was used for analysis. Individual data points are therefore not available, as the values represent the averaged results of each experimental condition.

The target Ct values of each sample were normalized to those of hGAPDH, which was considered a reference gene. The relative expression levels of all analyzed genes are expressed as fold change (2^ − ΔΔCt) compared to untreated control ADSCs. This approach allows normalized and reproducible comparison between groups while minimizing intra-assay variability, consistent with standard qPCR practice in REAC cellular studies^17–19^.

The primer sequences used are shown in Table 1.

**Table 1:** Primer sequences**.**

Primer name	Forward	Reverse
hGAPDH	GAGTCAACGGAATTTGGTCGT	GACAAGCTTCCCGTTCTCAG
Oct-4	GAGGAGTCCCAGGCAATCAA	CATCGGCCTGTGTATATCCC
Sox2	CCGTTCATGTAGGTCTCGGAGCTG	CAACGGCAGCTACAGCTAGATGC
NANOG	CATGAGTGTGGATCCAGCT	CCTGAATAAGCAGATCCAT
ACOT2	GAGGTCTTCACACTGCACCA	TCTTGGCCTCGAATGGTATC
LPL	CAGGATGTGGCCCGGTTTAT	GGGACCCTCTGGTGAATGTG
aP2	AGACATTCTACGGGCAGCAC	TCATTTTCCCACTCCAGCCC
PPAR-γ	AATCCGTCTTCATCCACAGG	GTGAAGACCAGCCTCTTTGC
UCP1	GTGGGTTGCCCAATGAATAC	TAAAAACAGAAGGGCGGATG

### Immunofluorescence

Immunofluorescence analysis was used to detect ASC-1 (marker of white adipocytes), PAT2 (marker of brown adipocytes), and TMEM26 (marker of beige adipocytes). All antibodies were previously validated for specificity and reactivity in human cell models in published REAC studies^17–19^, under identical culture and fixation conditions, confirming their reliability for the present work.

The immunostaining was performed according to standard protocols. Cells were fixed with 4% paraformaldehyde, permeabilized, and incubated with primary antibodies overnight at 4 °C. After washing, samples were incubated with Alexa Fluor–conjugated secondary antibodies, counterstained with DAPI, and visualized using a fluorescence microscope (Leica Microsystems, Germany).

### Statistical analysis

All data were analyzed using GraphPad Prism software (version 10.0). Before choosing the statistical test, data distribution was evaluated using the Shapiro–Wilk test, and homogeneity of variances was assessed using Levene’s test. When normality and homoscedasticity assumptions were met, parametric analyses (two-way ANOVA followed by Tukey’s post hoc test) were applied to evaluate the effects of treatment and differentiation medium. When these assumptions were not satisfied, non-parametric tests (Kruskal–Wallis and Wilcoxon signed-rank tests) were used instead. Each figure legend specifies the statistical test applied. Results are expressed as mean ± standard deviation (SD) for parametric data or as median with interquartile range (IQR) for non-parametric data, depending on the distribution. Statistical significance was set at p < 0.05.

## Results

### Stemness gene expression

The expression of stemness markers Oct-4, Sox2, and Nanog^29^ was significantly upregulated in ADSCs treated with REAC TO-RGN-AR compared with that in untreated controls and in cells cultured in the presence of adipogenic differentiation medium alone (DM). These results were consistent across six independent experiments, demonstrating the robustness of the findings.

Each measurement represents the mean of triplicate reactions for each condition, expressed as fold change relative to untreated controls. Importantly, this upregulation persisted even under differentiation-inducing conditions, which typically suppress the expression of stemness markers and promote lineage commitment.

**Fig. 1:**
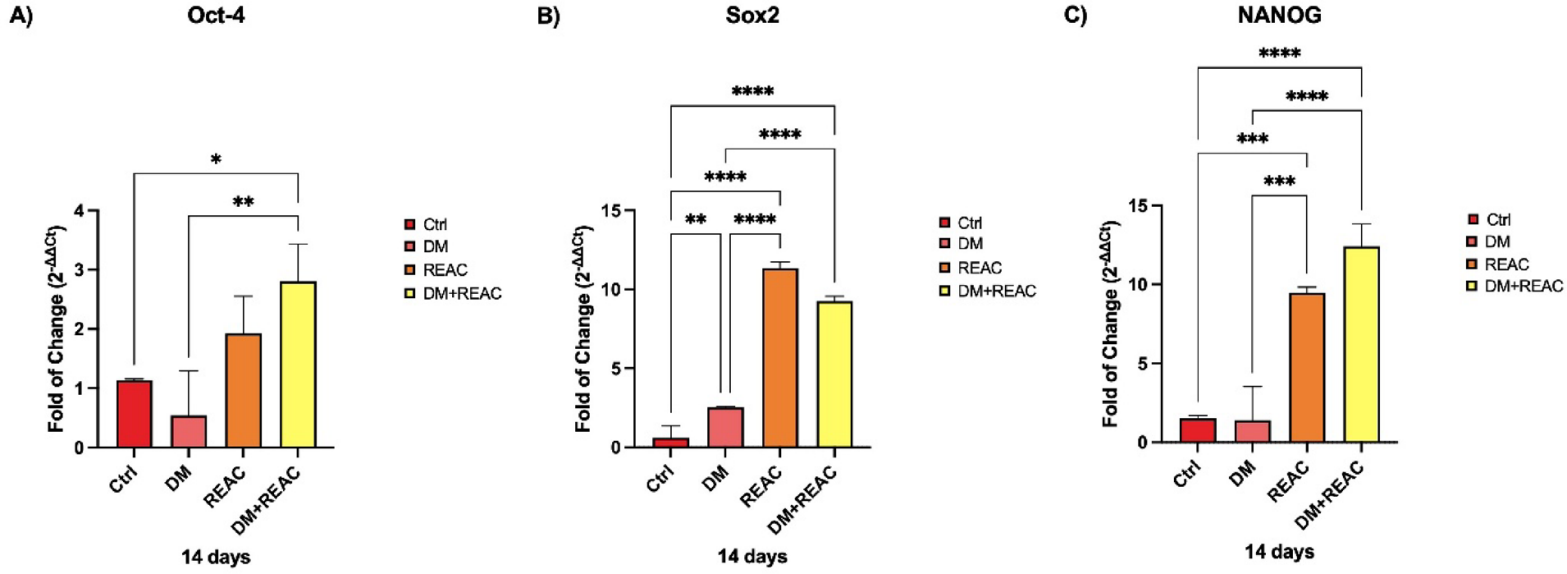
Expression levels of stemness-related genes Oct-4, Sox2, and Nanog in untreated ADSCs, ADSCs cultured with differentiation medium (DM), and ADSCs exposed to REAC TO-RGN-AR. Results are expressed as fold change relative to untreated controls.

This preservation of stemness indicates that REAC TO-RGN-AR treatment helps maintain cellular plasticity and self-renewal capacity, consistent with previous REAC studies on embryonic stem cells and human fibroblasts^17,18^, (Fig. 1).

### Dipogenic marker modulation

Adipogenic differentiation markers, including PPAR-γ^11^, LPL^31^, and ACOT2^30^, were significantly downregulated in REAC-treated ADSCs compared with those in cells exposed to adipogenic differentiation medium alone.

This reduction indicates that REAC TO-RGN-AR treatment suppresses the transcriptional program leading to adipogenic commitment and lipid accumulation. Importantly, cell viability remained above 95% in all experimental groups, confirming that the observed modulation was not associated with cytotoxic or inhibitory effects on cell proliferation.

The concurrent upregulation of UCP1^30^, a thermogenic gene characteristic of beige and brown adipocytes, suggests that REAC TO-RGN-AR promotes a shift toward a metabolically active and anti-inflammatory phenotype. This interpretation is consistent with previous REAC studies reporting similar transcriptional trends in adipose-derived stem cells^19^.

**Fig. 2:**
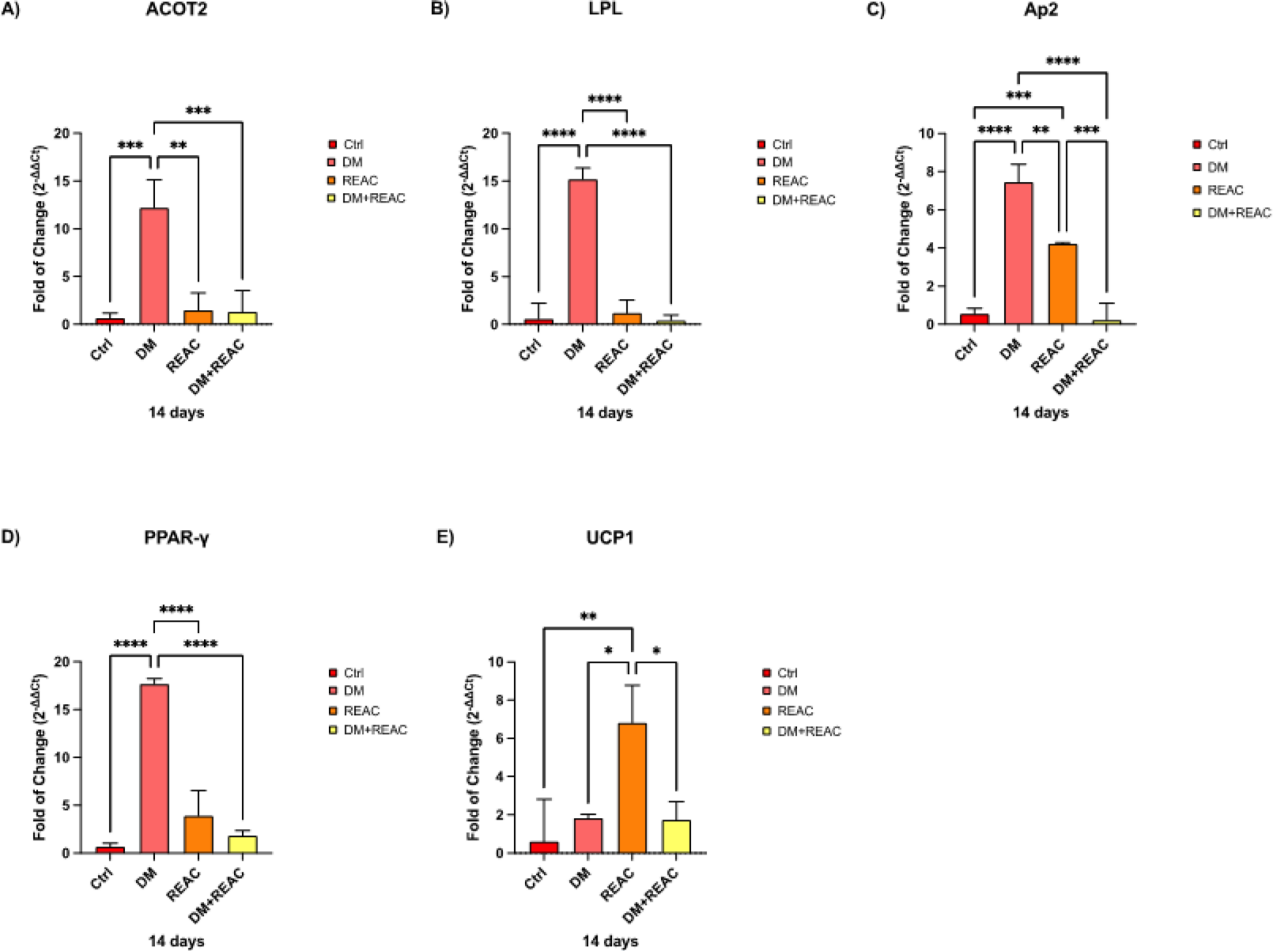
Relative expression of adipogenic and thermogenic genes (PPAR-γ, LPL, ACOT2, and UCP1) in ADSCs cultured in control medium, DM, or DM plus REAC TO-RGN-AR. Data are presented as mean ± SD from six independent experiments.

These findings confirm that REAC TO-RGN-AR treatment effectively suppresses adipogenic differentiation, affecting both the molecular and functional hallmarks of this process. Overall, the results indicate that REAC-induced bioelectrical modulation may help redirect ADSC fate away from white adipogenesis toward beige-like differentiation, a process potentially linked to AMPK/mTOR-regulated energy pathways. The selectivity of REAC TO-RGN-AR in modulating adipogenesis without affecting the overall viability or proliferation of ADSCs further underscores its therapeutic potential (Fig. 2).

### Phenotypic analysis

**Fig. 3:**
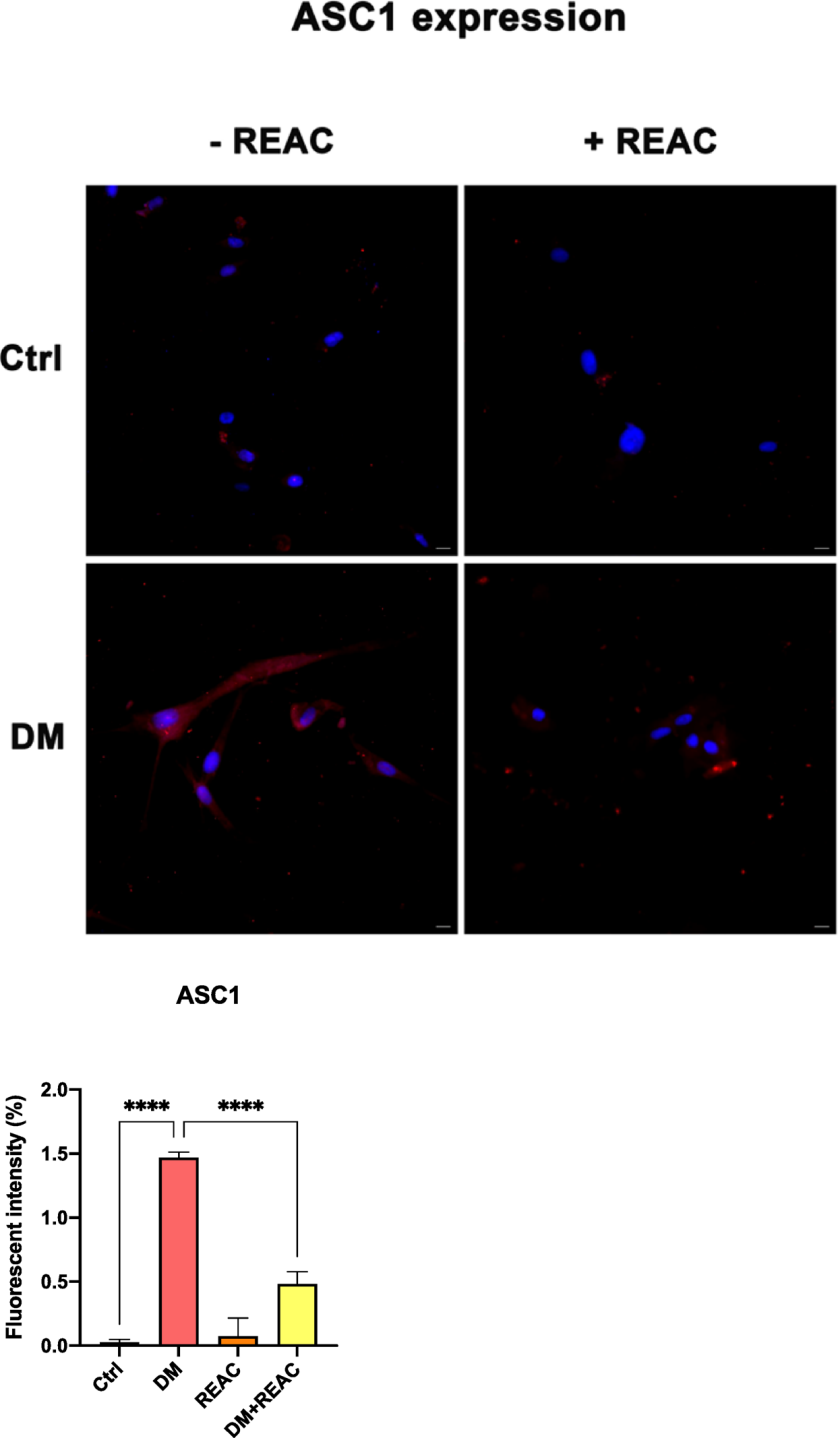
Representative immunofluorescence images showing ASC-1 expression in untreated ADSCs, ADSCs cultured in DM, and ADSCs exposed to REAC TO-RGN-AR. Nuclei were counterstained with DAPI. Scale bars: 40 µm. The fluorescence intensity was calculated using an image software analysis (ImageJ).

Immunofluorescence analysis revealed striking phenotypic differences between the REAC-treated and control groups. The cells treated with adipogenic differentiation medium alone presented high expression of ASC-1^33^, a white adipocyte marker (Fig. 3). REAC-TO-RGN-AR treatment also effectively suppressed the expression of ASC1 despite the presence of DM, (Fig. 3).

**Fig. 4:**
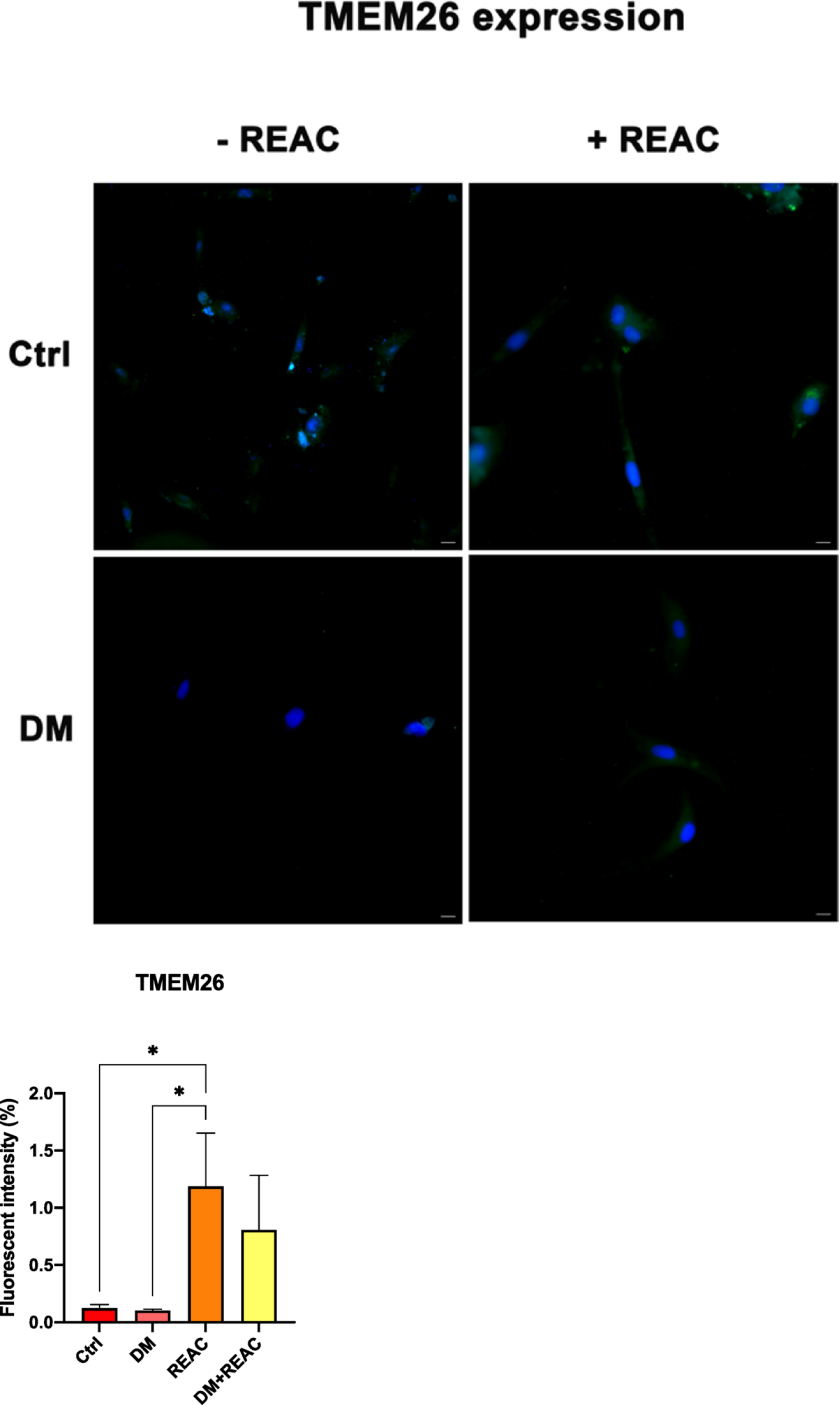
Representative immunofluorescence images showing TMEM26 expression in untreated ADSCs, ADSCs cultured in DM, and ADSCs exposed to REAC TO-RGN-AR. Nuclei were counterstained with DAPI. Scale bar: 40 µm. The fluorescence intensity was calculated using an image software analysis (ImageJ).

**Fig. 5:**
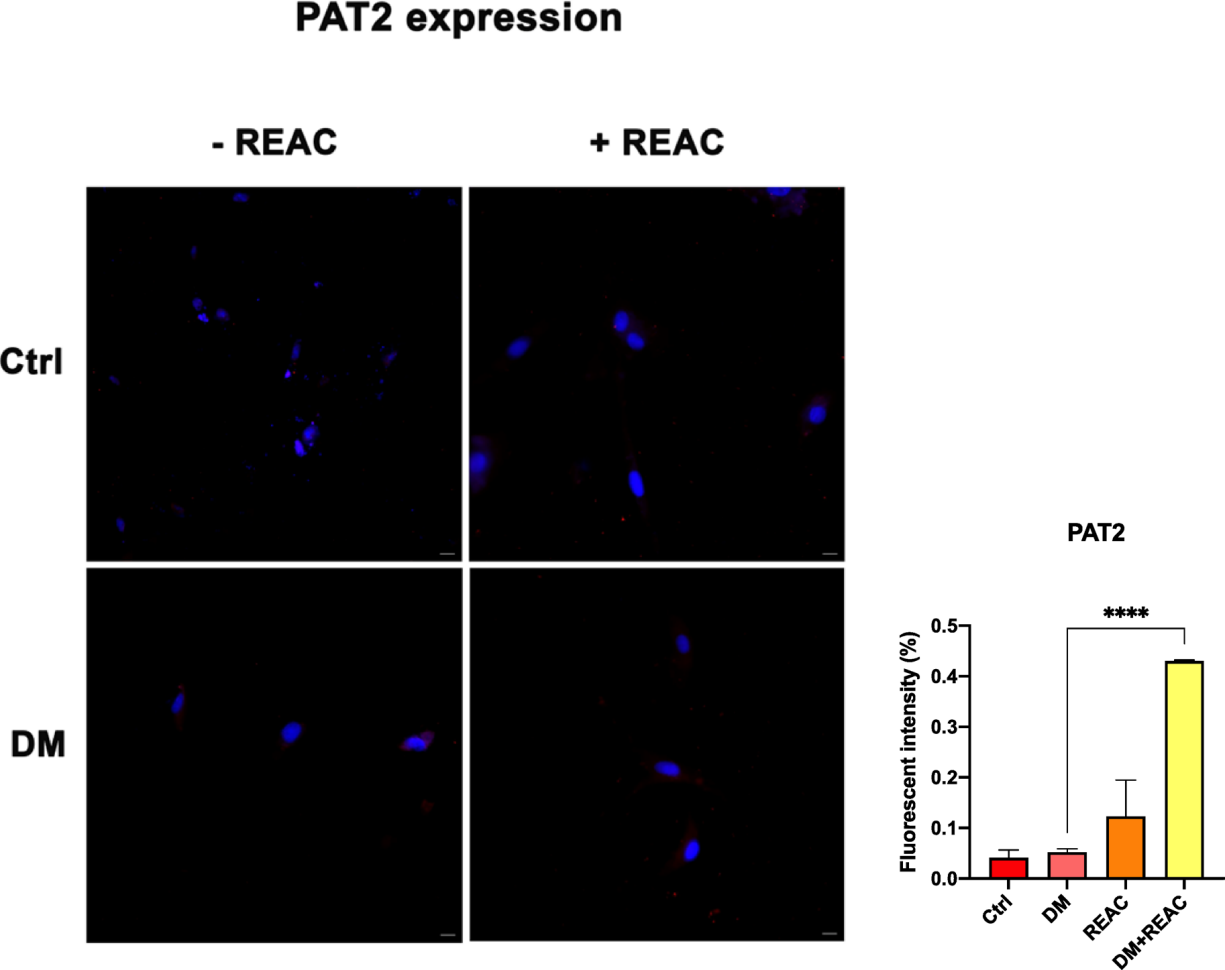
Representative immunofluorescence images showing PAT2 expression in untreated ADSCs, ADSCs cultured in DM, and ADSCs exposed to REAC TO-RGN-AR. Nuclei were counterstained with DAPI. Scale bar: 40 µm. The fluorescence intensity was calculated using an image software analysis (ImageJ).

Conversely, REAC-treated cells presented an increase in the expression of TMEM26^34^ (Fig. 4), a beige adipocyte marker, and a reduction in ASC-1 expression. The increased expression of TMEM26, together with PAT2^34^ (Fig. 4), a specific brown adipocyte marker, indicates a shift toward the beige adipocyte phenotype, which is associated with thermogenic and anti-inflammatory properties (Fig. 4 and Fig. 5).

The fluorescence intensity and distribution were consistently observed across independent replicates, and the representative images shown correspond to the most reproducible and well-defined patterns. Although quantitative morphometric analysis and co-staining with Bodipy were not performed, the qualitative consistency of the results supports their reliability. The antibodies used for ASC-1, TMEM26, and PAT2 were previously validated for specificity and localization in earlier REAC studies on human fibroblasts and adipose-derived stem cells^18,19^, confirming their suitability for the present work.

Importantly, no morphological alterations, abnormal proliferation, or cytotoxic effects were observed in any group, indicating that REAC TO-RGN-AR exposure did not compromise cell viability or phenotype integrity.

Cells treated with REAC TO-RGN-AR alone, without differentiation medium, presented negligible expression of all the markers, demonstrating the specificity of the treatment in modulating adipocyte lineage commitment. Taken together, these findings indicate that REAC TO-RGN-AR not only suppresses white adipogenic differentiation but also promotes the development of a metabolically favorable beige-like phenotype.

### Cell morphology

**Fig. 6:**
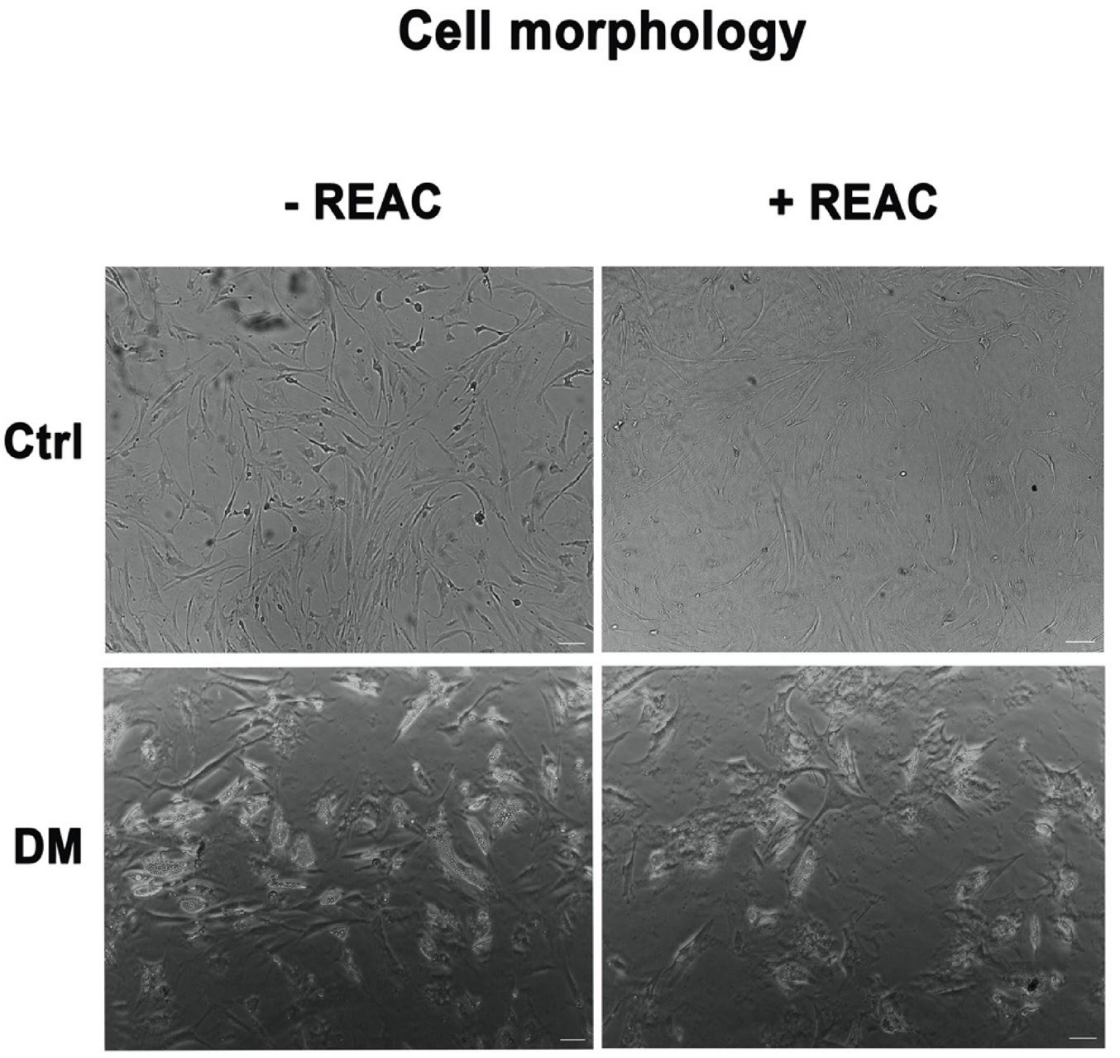
Analysis of REAC-treated ADSC morphology. Images were acquired under an inverted optical microscope. Scale bar 100 µm.

ADSC morphology was evaluated by optical microscopy. ADSCs cultured in the presence of the adipogenic differentiation medium and treated with REAC showed reduced mature adipocytes, as compared to ADSCs cultured in the presence of differentiation medium alone (DM – REAC), that showed an increased number of lipid droplets (Fig. 6). The same Figure showed that ADSCs treated with REAC in the presence of the basic growing medium maintained their typical morphology and proliferation rate, as compared to control untreated cells (Ctrl).

## Discussion

This study demonstrated that REAC TO-RGN-AR treatment modulates ADSC differentiation, preserves stemness, and promotes phenotypic shifts toward metabolically favorable beige adipocytes. By leveraging asymmetrically conveyed radio electric fields, this approach offers a novel strategy for influencing endogenous bioelectrical activity and holds promise for both foundational and translational applications in regenerative medicine.

The upregulation of stemness-related genes such as Oct-4, Sox2, and Nanog^29^ in REAC-treated ADSCs indicates a preserved cellular plasticity, even in the presence of differentiation-inducing stimuli. This property is fundamental to the regenerative potential of ADSCs and is consistent with previous studies demonstrating the influence of bioelectrical modulation on transcriptional networks involved in self-renewal and lineage specification^17–19^.

The concurrent downregulation of key adipogenic markers including PPAR-γ^11^, LPL^31^, and ACOT2^30^, alongside an increase in UCP1 expression, suggests a significant shift away from white adipocyte commitment. This is further corroborated by the immunofluorescence analysis, which revealed a decrease in ASC-1^33^ and an increase in TMEM26^34^ and PAT2 expression, markers indicative of beige/brown adipocyte phenotypes. Beige adipocytes are known for their thermogenic and anti-inflammatory capacities, making them particularly relevant in the context of therapies targeting metabolic dysfunction.

The modulation of adipogenic commitment observed here is particularly relevant in the broader context of metabolic disorders such as obesity, metabolic-associated liver disease (MetALD), and type 2 diabetes. In these conditions, the lipogenic and insulin-resistant environment enhances the adipogenic bias of ADSCs, impairing osteogenic and reparative capacities. Recent findings by Pinto et al. demonstrated that a high lipogenic state negatively affects osteogenic processes and tissue regeneration^35^, while Sanjabi et al. highlighted that lipid droplet hypertrophy in adipocytes plays a crucial role in metabolic dysregulation and insulin imbalance^36^. In light of these studies, the REAC TO-RGN-AR–induced suppression of adipogenic differentiation and promotion of beige adipocyte features may contribute to restoring metabolic balance and counteracting endocrine dysfunction.

Although this work was conducted entirely in vitro, the findings are consistent with prior in vivo studies involving REAC technology and provide a strong rationale for advancing to preclinical models. The influence of the native tissue microenvironment on ADSC behavior is well known, and future in vivo investigations will be essential to evaluate long-term outcomes and functional integration in metabolically altered tissues.

Donor metabolic status was not available, and this represents a limitation of the present study.

Protein-level confirmation and lipid staining (e.g., Oil Red O) were not performed and will be included in future analyses to strengthen these observations.

Future directions should also include the evaluation of ADSCs derived from donors with obesity or metabolic syndrome, which more accurately reflect the intended clinical applications. Establishing whether the observed bioelectrical modulation is equally effective in disease-relevant cell populations will be crucial to determining therapeutic applicability.

Furthermore, while the results point toward possible involvement of regulatory pathways such as AMPK and mTOR, both central to cellular energy sensing and mitochondrial dynamics, the present data do not allow for mechanistic confirmation. However, previous REAC studies have suggested that bioelectrical modulation may influence these same pathways, contributing to improved energy homeostasis, oxidative metabolism, and mitochondrial function. Dedicated mechanistic studies will be necessary to elucidate this relationship and confirm whether REAC-induced effects are mediated through AMPK/mTOR axis regulation.

This study also highlights areas for methodological development. For example, expanding the analysis to include dose–response and time-course studies would help define the exposure thresholds and temporal dynamics of response. Incorporating sham control conditions would improve the ability to exclude non-specific effects related to experimental handling. In addition, quantitative assessments of oxidative stress, viability, and potential off-target effects should be included in future investigations to comprehensively address the safety profile of this treatment. Further evaluation including additional beige markers such as CD137 and Ear2 may enhance the phenotypic characterization in future studies.

Overall, the ability of REAC TO-RGN-AR treatment to modulate adipogenic commitment, reduce markers associated with pathological lipid accumulation, and promote phenotypes linked to metabolic resilience reinforces the potential of endogenous bioelectrical modulation as a therapeutic frontier.

By restoring bioelectrical balance, REAC TO-RGN-AR may represent a promising strategy to mitigate the metabolic derangements associated with excessive adipogenesis and to improve the reparative capacity of ADSCs.

Beyond metabolic disorders, this approach may support broader regenerative goals, enhancing the functional integration of ADSCs in various tissue engineering^19^ contexts.

## Conclusions

REAC-TO-RGN-AR treatment is a novel, non-invasive approach to optimize ADSC differentiation. It preserves stemness and promotes beneficial phenotypes with anti-inflammatory and thermogenic properties. Further in vitro and in vivo studies are warranted to explore clinical applications in regenerative medicine and metabolic disorders.

## Data Availability

All data generated or analyzed during this study are included in this published article. For additional information or to request the dataset, please contact the corresponding author, Dr. Sara Cruciani.
